# Characterization of Chemical and Bacterial Concentrations in Floor Dust Samples in Southeast Texas Households

**DOI:** 10.3390/ijerph182312399

**Published:** 2021-11-25

**Authors:** Felica R. Davis, Hanan H. Ali, Jason A. Rosenzweig, Daniel Vrinceanu, Balaji Bhaskar Maruthi Sridhar

**Affiliations:** 1 Department of Environmental and Interdisciplinary Sciences, Texas Southern University, Houston, TX 77004, USA; f.davis7488@student.tsu.edu (F.R.D.); h.ali1524@student.tsu.edu (H.H.A.); 2 Department of Biology, Texas Southern University, Houston, TX 77004, USA; jason.rosenzweig@tsu.edu; 3 Department of Physics, Texas Southern University, Houston, TX 77004, USA; Daniel.Vrinceanu@tsu.edu; 4 Department of Earth and Environment, Florida International University, Miami, FL 33199, USA

**Keywords:** heavy metals, indoor dust, human exposure, geospatial, health risk

## Abstract

Indoor dust can be a major source of heavy metals, nutrients, and bacterial contamination in residential environments and may cause serious health problems. The goal of this research is to characterize chemical and bacterial contaminants of indoor, settled house dust in the Houston Metropolitan region. To achieve this, a total of 31 indoor dust samples were collected, along with household survey data, which were subsequently analyzed for elemental and bacterial concentrations. Microscopic and geospatial analysis was conducted to characterize and map potential hotspots of contamination. Interestingly Cd, Cr, Cu, Pb, and Zn concentrations of all 31 indoor dust samples were significantly enriched and exceeded soil background concentrations. Furthermore, As, Cd, Pb, and Zn concentrations in the dust samples were significantly correlated to the enteric bacterial load concentrations. Human health assessment revealed that cancer risk values via ingestion for Cd, Cr, and Ni were greater than the acceptable range. Of our 31 dust sample isolates, three Gram-negative and 16 Gram-positive pathogenic bacteria were identified, capable of causing a wide range of diseases. Our results demonstrate that both chemical and bacterial characterization of indoor dust coupled with spatial mapping is essential to assess and monitor human and ecological health risks.

## 1. Introduction

Indoor air pollution coupled with poor ventilation may cause serious problems, specifically for some women, children, and elderly who spend a great deal of time indoors. Most people spend approximately 80–95% of their time in indoor facilities breathing on average 10–14 m^3^ of air per day [1]. Worldwide, about 3.8 million premature deaths per year are attributed to indoor air pollution [2]. Adverse health effects such as cardiovascular deaths, asthma, nervous system disorders, and stunted growth are among the many conditions associated with indoor air contamination [2,3,4]. Indoor contaminants often get suspended in air, adhere to particulate matter, and then settle as dust covering furniture, floors, and other objects [5].

Among the most prominent contaminants in indoor dust are heavy metals, which are non-degradable, toxic, carcinogenic, and have an acute or chronic impact on human health [3,4,6,7]. Household consumer products, electrical appliances, carpets, building materials, and other products serve as extensive sources of heavy metals in indoor dust [5,6,8]. External sources of dust such as soil, road dust, and industrial and vehicular particulates often enter homes via the airborne route or are carried by inhabitants [7]. Indoor floor dust acts as a major sink for airborne pollutants including a wide variety of organic and inorganic chemical contaminants in addition to bacterial, fungal, and viral contaminants [4]. The quality of indoor dust often depends on the natural and anthropogenic sources of contamination, surrounding environment, economic development, and outdoor air quality. Hence, indoor floor dust can serve as a key indicator of indoor contamination and human health risks of a geographical region [9]. 

Exposure to indoor dust can occur through inhalation, ingestion, and dermal routes [10]. Children are the most vulnerable group affected by exposure to heavy metals in dust [7] because of frequent hand-to-mouth movement and crawling on the floor. In humans, bioaccumulation of Cd causes kidney injuries, tumors and hepatic dysfunction, poor reproductive capacity, and hypertension [11], while As causes cardiovascular disease, skin cancer, developmental anomalies, neurologic, neurobehavioral, and hematologic disorders [12]. Accumulation of Cr affects the respiratory tract [13] while Pb toxicity can affect nearly every organ and system in the body, slows growth, causes anemic seizures, and behavioral and learning difficulties in children [14]. Consequently, USEPA revised the dust lead clearance levels (DLCL) from 40 μg/ft^2^ and 250 μg/ft^2^ to 10 μg/ft^2^ and 100 μg/ft^2^ for floors and windowsills, respectively [14].

Various global studies have been conducted on the elemental and bacterial concentrations in indoor floor dust collected from households and other indoor environments in urban areas [5,7]. However, few are conducted in the United States and none were located in the Houston metropolitan region. The Houston metropolitan region suffers from poor air quality on account of industrial point sources such as: petroleum refineries, on-road emissions from motor vehicles and off-road emissions from ships, airplanes, freight trains, and construction activities [15,16]. Therefore, the objectives of the study are: (1) to identify and determine the selected elemental and bacterial concentrations in representative indoor dust samples from Harris County and surrounding areas, (2) to assess the effect of indoor habitat on the dust composition and characterize the samples using microscopy, and (3) to analyze the spatial distribution of contaminants and assess human health risks from indoor dust exposure.

## 2. Materials and Methods

### 2.1. Study Area and Sample Collection

**Figure 1:**
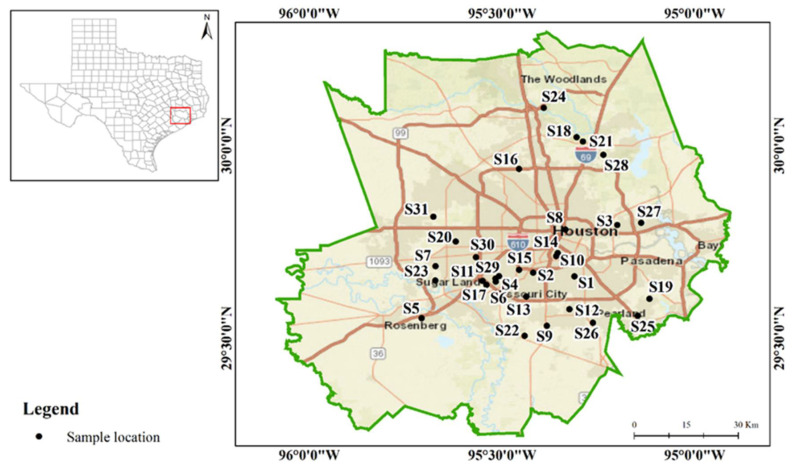
The study area and the sampling locations within Southeast Texas.

Indoor house dust samples were collected from 31 residences (1 sample per residence) using vacuum cleaners during the winter season of 2019 (Figure 1), when homes are most tightly sealed. Dust samples were randomly collected from volunteer participants who reside in the Greater Houston area. The selected residences are not near any industrial, mining, or other major sources of pollution. Information about household attributes such as type of residential unit, flooring, cooling, and heating units, number of occupants, and vacuuming frequency was collected through a systematic survey. Supplemental data (Table S1) provide specific information about the participants and activities occurring in the home that may contribute to elemental and bacterial dust contamination. All households were in the greater Houston region, within the boundaries of Harris County (*n* = 22), Fort Bend County (*n* = 4), Brazoria County (*n* = 4), and Montgomery County (*n* = 1). The dust samples were air dried and then sieved through a 63 µm sieve.

### 2.2. Chemical Analysis

Approximately 0.5 g of dust sample from each replicate was measured and microwave (Mars 6, CEM, Matthews, NC, USA) digested in 10 mL of HNO_3_ using the EPA 3050B [17] digestion method for soil. The digested samples were analyzed for elemental concentrations by using Inductive Coupled Plasma Mass Spectrometry (ICP-MS, Agilent 7500 Series, Santa Clara, CA, USA).

### 2.3. Statistical and Spatial Analyses

Statistical analysis of the data was performed using Microsoft Excel 2019 and IBM SPSS Statistics Version 27. A 2-tailed Pearson correlation coefficient test was used to measure the degree of correlation between the elemental concentrations and household attributes where 0.40–0.69 was considered as moderate correlation, 0.70–0.89 as strong correlation, and 0.90–1.00 as very strong correlation [18]. Spatial distribution maps of heavy metals were created using ArcGIS 10.8 software to analyze and map the possible hotspots of contamination.

### 2.4. Scanning Electron Microscope (SEM) and Energy Dispersive Spectrometer (EDS) Analysis

Selected dust samples were analyzed to examine the physical and chemical characteristics of the dust particles using a Scanning Electron Microscope (Prisma E Color SEM, Thermo-Scientific, Waltham, MA, USA). Dust samples were immobilized on double-sided carbon tape, mounted on a SEM to determine size, shape, and other morphological characters. The elemental composition of the identified particles was analyzed using X-ray Diffraction (XRD) analysis (Pathfinder X-ray Microanalysis software).

### 2.5. Heavy Metal Enrichment

Enrichment factor (EF) was calculated for individual heavy metals by using the equation,

EF = (*C_i_*/*C_Al_*)/(*C_Bi_*/*C_BAl_*), (1)

where *C_i_* is the concentration of the element of interest in the dust sample, *C_Al_* is the concentration of the reference element (Al) in the dust sample, *C_Bi_* is the background value of the corresponding element, and *C_BAl_* is the background value of reference element. The reference element used in this study was Al, and the background values of elements in Texas soils were used as reference data [19]. The EF value close to one indicates natural level, while values greater than 10 indicates contamination from anthropogenic sources [7]. EF quantifies how much an element in a sampling medium has increased relative to its average natural abundance [20]. Enrichment levels are divided into five categories where EF values less than 2 indicate low to minimal enrichment, 2–5 is moderate enrichment, 5–20 is significant enrichment, 20–40 is very high enrichment, and greater than 40 is extremely high enrichment [21].

### 2.6. Health Risk Assessment

Health risks from ingestion exposure were calculated for younger children (YC) of less than 6 years age, older children (OC) of 6 to 18 years age, and adults (A) of 18 years and older age using the equation:

ADD_ing_ = *C* × *IgR* × *EF* × *ED* × *CF/BW* × *AT* (2)

where *C* is the concentration of the heavy metal of interest in dust (mg/kg); *IgR* is the ingestion rate of dust (mg/day); *EF* is the exposure frequency (365 days); *ED* is the exposure duration (YC: 6 years, OC: 12 years, A: 30 years); *CF* is the conversion factor (1 × 10^−6^ kg/mg); *BW* is body weight (YC: 15 kg, OC: 48 kg, A: 70 kg); and *AT* is the average lifetime (365 × *ED* and 365 × 70 for non-carcinogenic and carcinogenic, respectively) [22]. Dermal exposure risk is calculated using the equation:

ADD_dermal_ = *C* × *SA* × *AF* × *ABS* × *EF* × *ED* × *CF/BW* × *AT* (3)

where *SA* is the exposed skin area (YC: 2336 cm^2^, OC: 4591 cm^2^, A: 6034 cm^2^); *AF* is the skin adherence factor for soil/dust (YC: 0.2 mg/cm^2^/day, OC: 0.2 mg/cm^2^/day, A: 0.07 mg/cm^2^/day), and *ABS* is the dermal absorption factor from the dust [22]. Inhalation risk is estimated using the equation

ADD_inhale_ = *C* × *IhR* × *EF* × *ED/PEF* × *BW* × *AT* (4)

where *IhR* is the inhalation rate (20 m^3^/day); and *PEF* is particulate emission factor (1.36 × 10^9^ m^3^/kg) [22]. To estimate non-carcinogenic risks of HMs in dust, the hazard quotient (HQ) was calculated by dividing the ADD for exposure routes by a chemical-specific reference dose (RfD) [23]. The HQ from ingestion, dermal, and inhalation exposures is summed to obtain the hazard index (HI). HI values greater than 1 indicate potential non-carcinogenic effects, while values less than 1 suggest no significant risk of non-carcinogenic effects [24].

Cancer risk (CR) is the probability that an individual will develop cancer if exposed to a chemical during a lifetime of 70 years [25]. The cancer slope factor (CSF) is used to estimate cancer risk associated with exposure to a carcinogenic or potentially carcinogenic substance. Carcinogenic risks to humans from ingestion, dermal, and inhalation exposures are estimated by multiplying the average daily doses (ADD) by the corresponding cancer slope factor (CSF) by the equation [23],

LCR = *ADD* × *SF* (5)

The total lifetime cancer risk (TLCR) is the sum of all LCRs calculated for ingestion, dermal contact, and inhalation. Cancer risk estimates are classified as follows: very low (TLCR ≤ 10^−6^), low (10^−6^ < TLCR ≤ 10^−4^), moderate (10^−4^ < TLCR ≤ 10^−3^), high (10^−3^ ≤ TLCR < 10^−1^), and very high (TLCR ≥ 10^−1^) [26]. The EPA considers values in the range of 10^−4^ to 10^−6^ as acceptable cancer risk.

### 2.7. Bacterial Analysis

Total and enteric bacterial load concentrations in dust samples were determined by using our previously published methods [27]. In short, the broad medium Luria–Bertani (LB) agar (BD Difco™), while the selective and differential MacConkey agar (Difco^®^) medium was used to enrich for enteric bacteria. Twenty-one representative down-selected colony isolates from both LB and MacConkey plates were subjected to Gram-staining and oxidase (BD oxidase reagent dropper catalog #261181) and catalase testing with 3% H_2_O_2_.

Next, the twenty down-selected colony isolates were identified by the BIOLOG GEN III identification system (BIOLOG, Hayward, CA, USA). Pure bacterial cultures were suspended in an inoculating fluid (IF-A GEN III Cat #: 72401) to a specified density (~0.2 OD_600 nm_) using the turbidity meter (BIOLOG TM). Bacterial suspensions (100 µL) were pipetted into each well of the micro-plate (GEN III Cat #: 1030) and incubated at temperatures of either 37 °C for Gram-negative or 32 °C for environmental isolates for a minimum of 24 h. The micro-plate was then read with the BIOLOG Micro Station system and compared to the database for the identification of the microbial organism species.

To confirm our BIOLOG identifications, colony PCR reactions were set up as previously described [27]. Ribotyping using Sanger sequencing was conducted by Lone Star Labs Inc. (Houston, TX, USA). The universal forward primer (27F) (AGAGTTTGATCCTGGCTCAG) was diluted to 5 pmol and added to 5 μL of the PCR Reaction sample. A Quat-iT PicoGreen dsDNA Assay kit was used to measure the final concentration at A260/A280 ratio. Agilent 2100 Bioanalyzer device (Agilent Technologies) was used to estimate the library (sample) size by using Agilent DNA 7500 kit. The cluster densities were optimized by quantifying the libraries using a fluorometric method that utilizes dsDNA binding dyes. Using Geneious version 2021.0 software, which displays the evolutionary relationships between species, a phylogenetic tree was created.

## 3. Results

### 3.1. Chemical Analysis

The average heavy metal, nutrient, and bacterial concentrations categorized, based on the characteristics and habitat of the household sampling locations, are given in Table 1 and Table 2. Mean concentrations for Cd, Cu, Ni, Pb, and Zn (Table 1) in all the sampling locations were above background concentrations occurring naturally in Texas soils. Among the habitat category of home type, the Al concentrations were significantly (*p* < 0.05) higher in apartment houses, while the Cu concentrations were significantly (*p* < 0.05) higher in single-family houses compared to others (Table 1). The concentration of As, Cd, Fe, Ni, Pb, and Zn were higher in single-family houses compared to apartments (Table 1). Houses over 30 years old showed a significantly (*p* < 0.05) higher concentration of Cd, Pb, and higher concentrations of Al, As, Cu, Ni, and Zn compared to the houses younger than 30-years old (Table 1).

Regarding flooring type, houses with no carpet have significantly (*p* < 0.05) higher concentrations of Cu, Pb, and Zn compared to the partial and fully carpeted houses (Table 1). As, Cd, Cu, Pb, and Zn concentrations were higher, and Al was significantly (*p* < 0.05) higher in houses with pets compared to houses with no pets (Table 1). Concentrations of Cd were significantly (*p* < 0.05) higher and As, Cr, Cu, Fe, Ni, Pb, and Zn concentrations were also higher in houses using gas as their primary source of heating compared to electricity (Table 1).

Surprisingly, concentrations of Mn, and enteric bacteria in the dust samples of single-family houses were significantly (*p* < 0.05) higher than in multi-family households (Table 2), despite fewer people residing within. For reasons not well understood, houses under 10 years old were significantly (*p* < 0.05) higher in total bacteria and enteric bacteria concentrations (Table 2). Additionally, concentrations of Na, Mg, K, Ca, and total bacteria were higher in the dust single-family household dust samples as well (Table 2). Carpeted houses showed higher concentrations of Na, total and enteric bacteria, while no trends were observed for other elements (Table 2). Not surprisingly, both total and enteric bacterial concentration loads were higher in households containing pets, while the Na, Mg, Ca, and Mn concentrations remained higher in households with no pets (Table 2). Concentrations of K, Ca, Mn, and enteric bacterial concentrations were higher in houses equipped with gas as a source of heating compared to electricity (Table 2).

Inter-elemental relationships can provide helpful information regarding possible contamination sources of heavy metal contamination in indoor dust. Pearson correlation coefficients of heavy metals (Table 3) revealed strong (r = 0.7–0.89) and highly significant (*p* < 0.01) positive correlations between Zn and Cu, Mn and Fe, Zn and Fe, total and enteric bacteria, Zn with Cu and Fe, and Mn with Fe (Table 3). Moderate (r = 0.4–0.69) highly significant (*p* < 0.01) positive correlations were found for: (1). Al with Cr and Pb, (2). As with Cd, Cr, Ni, and Pb, (3). Pb with Cd and Cr, (4). Cu with Fe, Ni, Zn, and K, (5). Fe with Ni, Zn, K, and Mn, (6). Ni with Zn and Mn, (7). between Zn and Mn, and (8). between Mg and Ca (Table 3). Total bacterial concentrations were significantly (*p* < 0.05) correlated with As, Ni, and K (Table 3), while enteric bacterial loads were significantly (*p* < 0.01) correlated with As, Cu, Ni, and K. Also, significant (*p* < 0.05) correlation was observed with Fe, Pb, and Zn (Table 3). Conversely, Na showed a significant negative correlation with Al, Cr, and Pb concentrations (Table 3).

**Figure 2:**
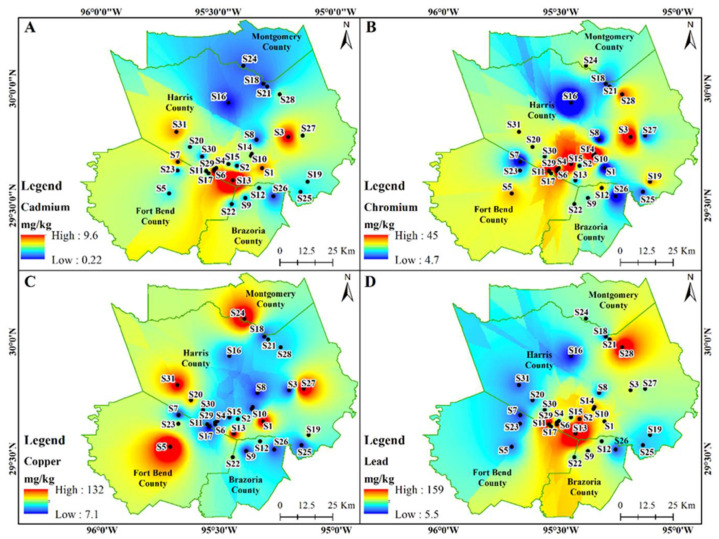
The spatial distribution of Cd (**A**); Cr (**B**); Cu (**C**); and Pb (**D**) concentrations in the indoor dust samples.

Spatial distribution of heavy metals in dust samples exhibited varied patterns. The concentration of Cd exceeded the ecological screening value (ESV) limits (0.36 mg/kg) for all sample locations (except S16 and S18), of which S13 (9.6 mg/kg) had the highest level of contamination followed by S6 (8.8 mg/kg) and S3 (3.9 mg/kg) (Figure 2A). The Cr concentrations exceeded ESV limits (23 mg/kg) at various sites including S28, S3, S14, S10, S6, S29, S11, and S15 locations ranging from 24–45 mg/kg (Figure 2B). Most sample sites exceeded the standard value of Cu (28 mg/kg) with sites S31, S13, S27, S1, S24, and S5 being over 3 times the ESV limit (101–132 mg/kg) (Figure 2C).

**Figure 3:**
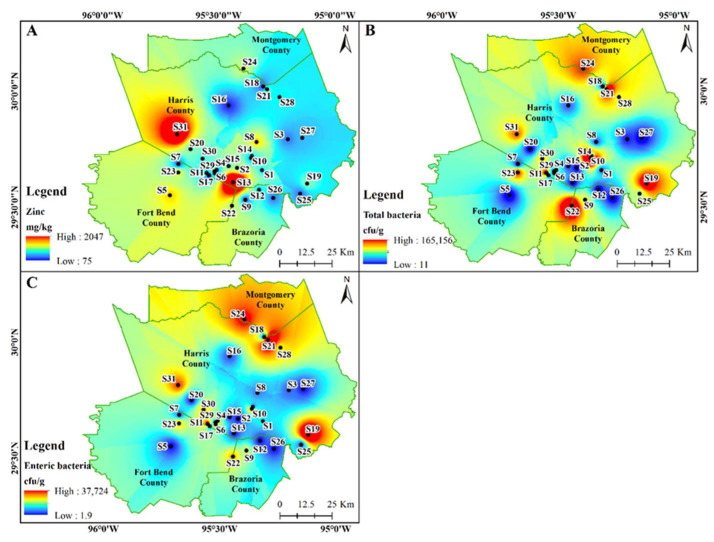
The spatial distribution of Zn (**A**); total (**B**); and enteric bacteria (**C**) in the indoor dust samples.

Alarmingly, 27 of the 31 sample sites had Pb concentrations above acceptable limits (11 mg/kg) with sites S2, S28, S6, S11, and S13 being the highest at 62–159 mg/kg (Figure 2D). All 31 sample sites in our study surpassed ESV limit of 46 mg/kg for Zn with sites S31 and S13 having the highest concentrations at 1166 mg/kg and 2050 mg/kg, respectively (Figure 3A). Total bacteria concentrations were found to be higher at sites S19, S21, S22, and S10 (Figure 3B), while S31, S24, S11, S19, and S21 locations showed high enteric bacteria concentrations (Figure 3C).

### 3.2. Scanning Electron Microscopy (SEM)

**Figure 4:**
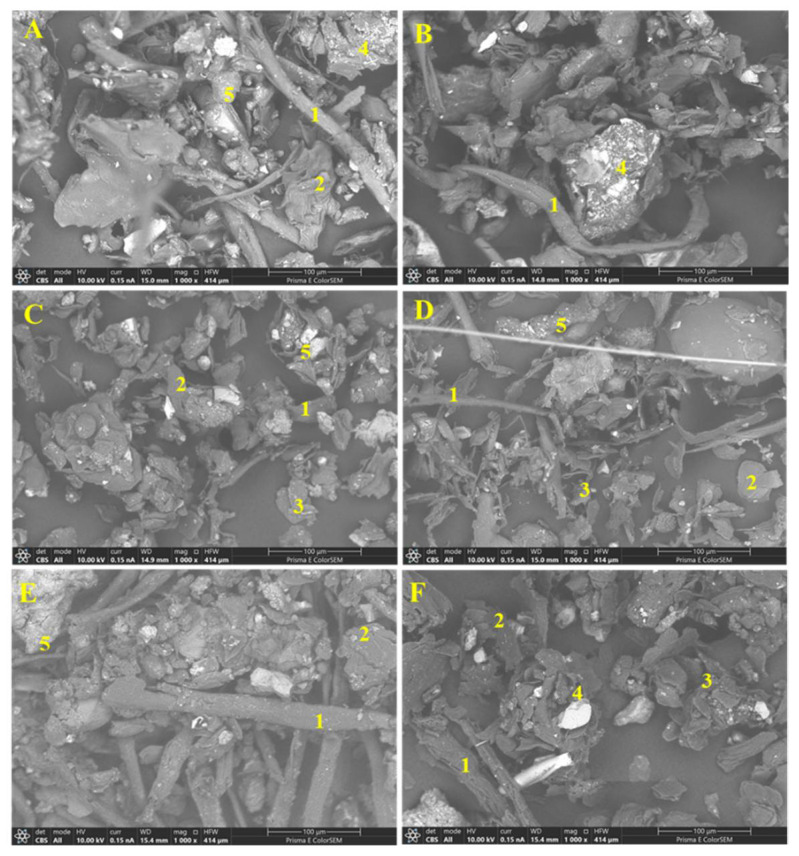
Scanning Electron Microscope (SEM) images showing the structure and composition of the indoor dust samples obtained from S10 (**A**); S19 (**B**); S21 (**C**); S22 (**D**); S23 (**E**); and S24 (**F**) sampling locations. The image displays fiber (1), skin (2), sand (3), calcium (4), and sodium (5) salts.

The SEM images revealed particles of fiber, skin, sand, calcium, and sodium salts within indoor dust samples (Table 4 and Figure 4). Major elements found in the particles include C, O, Na, Al, Si, S, Cl, K, and Ca with carbon and oxygen having the highest percentages in all particles (Table 4).

SEM images of representative dust samples from sites S10, S19, S22, S21, S23, and S24 located in the central, eastern, southern, western, and northern parts of the Houston-Galveston study area were compared (Figure 4). The irregular shapes and sizes of the dust particles vary from compact and rounded to thin flakes, fibrous, angular, and aggregates (Figure 4). The differences in size, morphology, and agglomeration were observed based on the particle type (Figure 4). Among the dust particles, the fibers had a thin, long, and elongated shape; the skin particles appeared as thin, irregular flakes while the dust particles appeared as spherical or irregular aggregates. Salt particles appeared as angular, crystalline aggregates (Figure 4). The size, shape, and other physical properties of the respirable dust particles affect the availability and distribution of chemicals associated with dust and their impact on human health.

### 3.3. Enrichment of Measured Metals

**Figure 5:**
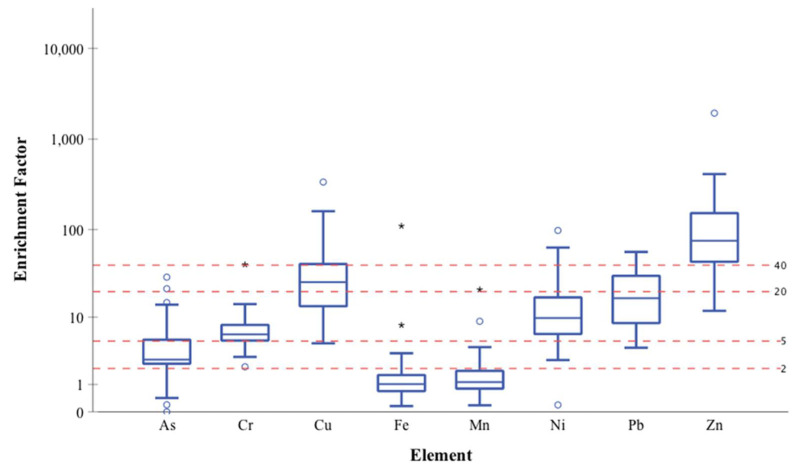
Boxplots for the enrichment factors (EF) of heavy metals in the 31 floor dust samples from the Harris County and surrounding areas relative to the local natural soil (Al was chosen as reference element). * The top and bottom of each box represent 75th and 25th percentiles, respectively, line across inside of each box represents median, and asterisk beyond whiskers are outliers.

The degree of enrichment of metals in dust samples of individual metals was estimated (Figure 5). Based on the enrichment factor (EF) calculations, enrichment values decreased as follows: Zn > Cu > Pb > Ni > Cr > As > Fe > Mn. Fe and Mn enrichment was relatively low (EF < 2) indicating minimal enrichment. Arsenic is moderately enriched with the EF value between 2 and 5, while the EF value for Cr, Ni, and Pb was between 5 and 20 representing significant enrichment in the indoor environment (Figure 5). Cu and Zn had EF values greater than 20 indicating very high to extreme enrichment (Figure 5).

**Figure 6:**
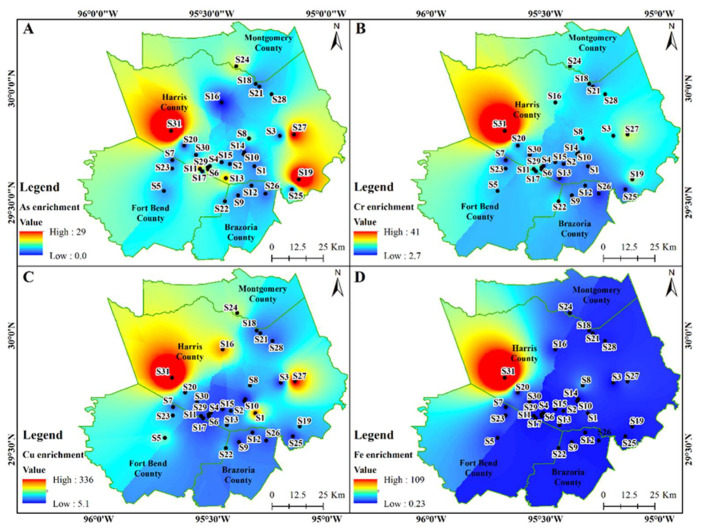
Spatial distribution of As (**A**); Cr (**B**); Cu (**C**); and Fe (**D**) enrichment in the indoor dust samples in Southeast Texas.

**Figure 7:**
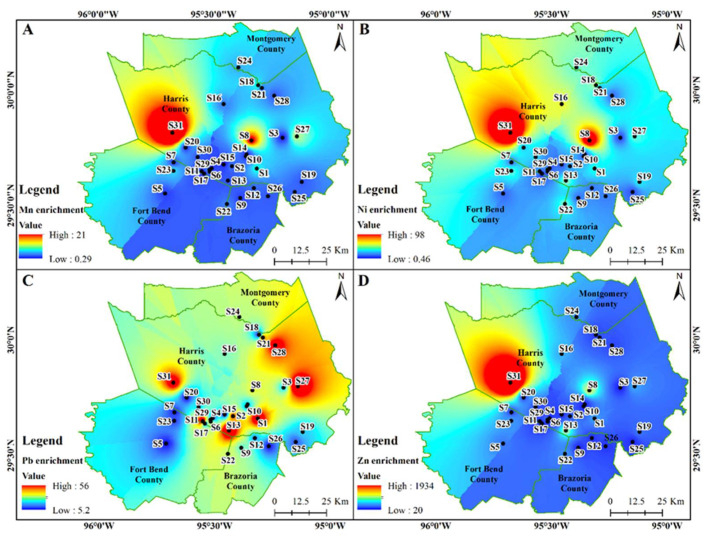
Spatial distribution of Mn (**A**); Ni (**B**); Pb (**C**); and Zn (**D**) enrichment in the indoor dust samples in Southeast Texas.

The As, Cr, Cu, Fe, Mn, Ni, and Zn were all highly enriched (20 < EF < 40) to extremely high enriched (EF > 40) for sample location S31 in the western part of Harris County (Figure 6 and Figure 7). Generally, heavy metal enrichment in indoor dust was higher in Harris County relative to the surrounding counties. Alarmingly, Pb enrichment showed a diverse spatial pattern within Harris County where S13, S11, S27, S1, S28, and S31 all had EF > 40 indicating extremely high enrichment (Figure 7C).

### 3.4. Health Risk Assessment Results

Seven priority heavy metals (As, Cd, Cr, Cu, Ni, Pb, and Zn) were included for estimating the human health risk for children and adults because of their strong toxicity potential. Results revealed that the hazard indices were less than the acceptable or tolerable risk level of 1, indicating no significant non-carcinogenic risks from exposure to heavy metals in household dust. However, the total lifetime cancer risk (TLCR) for children under the age of 6 years exceeds the EPA’s acceptable cancer risk range of 10^−6^ to 10^−4^ (Table 5). 

The cancer risk values via ingestion (LCR ingestion) for Cd, Cr, and Ni was 1.18–4.52, 1.12–1.67, and 1.30–1.98 times greater than acceptable cancer risk range (Table 5). The TLCR via all exposure routes exceeded the acceptable range by 1.21–4.52 times for Cd, 1.15–1.71 times for Cr, and 1.33–2.02 times for Ni in all counties except Fort Bend (Table 5). In contrast, the LCR and TLCR for As was within the acceptable range (Table 5).

### 3.5. Bacteria Isolate Results

From our total and enteric loads, 14 representative colony isolates were down selected for further characterization and identification (Table 6). Using the BIOLOG Microstation, 3 Gram-negative and 16 Gram-positive bacteria were identified (Table 6). 

**Figure 8:**
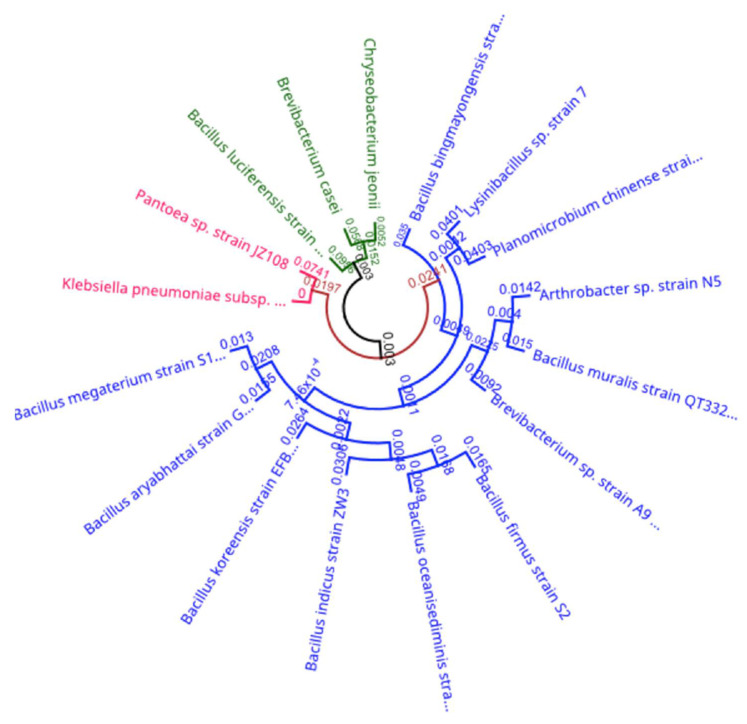
The Phylogenetic tree for the indoor dust bacteria species isolated by 16S ribotyping RNA sequencing.

A phylogenetic tree displaying the evolutionary relationships among the isolated bacterial species from indoor dust samples was created (Figure 8). Notable among the Gram-positive isolates are the spore forming *Bacillus* spp. Gram-negative isolates are indicated in and included the pathogenic *Klebsiella aerogenes* (Figure 8).

## 4. Discussion

Concentrations of Cd, Cu, Ni, Pb, and Zn in our indoor dust samples exceeded the background soil limits (Table 1). The higher concentration of metals in indoor dust over outdoor soil is attributed to the ability of organic rich indoor dust to accumulate metals over soil [5]. The common sources for Cu in the house dust include building material and products such as pipes, electrical appliances, wires, and treated wood; Pb can be sourced to cheap metal jewelry, plastic toys, paints, and solder, Ni from stainless steel debris, Zn from rubber carpets, household appliances such as refrigerators, air conditioners and washing machines, and Cd from various plastics and batteries [4,5]. Other sources of metals in indoor dust include cooking, fuel combustion, smoking, candles, incense burning, shedding of fibers, furniture dust, and infiltration of outdoor particles [5].

The higher concentration of Al in multi-family dwellings is likely from the ubiquitous use of Al wiring on older multi-family buildings. About 1.5 to 2 million single-family homes, mobile homes, and multiple-family dwelling built between 1965 and 1971 were wired with Al [29]. Various other sources of Al in household dust include radios, toasters, electrical wiring, refrigerators, and personal care products [30]. Elevated levels of Pb and Cd in older homes of over 30 years (Table 1) could be due to deteriorating lead-based paint used in houses built before 1978 [31]. Houses with uncarpeted floors showed a higher concentration of Zn, Pb, and Cd than both partial and 100% carpeted dwellings. This can be attributed to the elevated levels of Pb, Cd, and Zn in the vinyl and laminate floorings [32]. Significant Cd concentrations in houses heated by gas is likely from the burning of fossil fuels [33].

The concentrations of macro elements such as Na, Mg, K, Ca, and Mn (Table 2) were higher than the heavy metals in indoor dust samples (Table 1). The source of these elements includes various minerals such as quartz, albite, calcite, and dolomite, which are predominantly distributed in the outdoor dust of both urban and rural regions [9]. The reasons for high concentration of enteric bacteria, Mn, and other elements in single-family homes over multi-family homes remains unclear to us. Perhaps, it can be attributed to carry over of outdoor dust by increased human activities and living style [9]. However, dust samples from houses with pets showed higher total and enteric bacterial concentrations (Table 2). This confirms that the bacterial and fungal richness and biodiversity is higher in the indoor dust of houses with pets [34]. Cu, Fe, Zn, Pb, and Ni were significantly correlated (Table 3) with each other indicating that these elements may have derived from the same or closely related sources. Cu, Fe, Zn are related to wear parts of alloys, used in machinery, lubricants, building materials, appliances, and wood products [4].

The concentrations of Cd, Cr, Cu, and Pb were higher in the sample locations from urban centers (Figure 2) compared to the sub-urban regions. Differing degrees of urbanization, vehicular traffic, road network, and economic development impacts the indoor and outdoor dust quality [9]. The physical and chemical characteristics of our indoor dust samples were found to be different in the regions of rapid and slow development [9]. In addition, indoor dust sampled from locations near major roadways was highly correlated to the outdoor particle pollution related to traffic [2,35]. We observed concentration of Zn in indoor dust to be higher in urban centers while the total and enteric bacteria concentrations were elevated in the suburban regions (Figure 3). Perhaps this is because, apart from the outdoor air, bacterial concentrations in indoor dust depend on ventilation, occupant behavior and activities, pets, and building materials, etc. [35]. Since dust samples were collected during the winter when homes are tightly sealed, we expected higher elemental concentrations. If additional samples were collected in the summer season, concentrations would be lower compared to the winter season as ventilation helps to remove or dilute indoor airborne contaminants.

The morphological and elemental composition of the dust samples, as determined by SEM and X-ray diffraction, revealed that it consisted of mainly carbon and oxygen in the particles identified as fiber, skin, sand, and salts (Table 4; Figure 4). The shape and size of the dust particles can also be used to identify the source of the dust. Vehicular area minerals have more irregular shapes compared to the industrial and residential area sources [36]. Smaller dust particles possess higher air mobility, longer residence time in the atmosphere, and, hence, possess higher inhalation and ingestion risk affecting human health [37,38]. Several studies have suggested that the bioaccessibility and cytotoxicity of metals depend on their adsorption to various dust particles [38].

The Enrichment Factor (EF) of As, Cr, Cu, Pb, and Zn was higher than one, indicating considerable enrichment in the household environment relative to their natural abundances (Figure 5). The EF of Cu and Pb exceeded 20, indicating that the abundance of these elements was more concentrated in indoor dust than natural occurrence (Figure 5). The Zn, Pb, and Cu showed the high EFs values in indoor dust at 166, 47, and 22, respectively (Figure 5), suggesting anthropogenic sources were contributing to their release into the ambient environment. Vehicular traffic, road dust, electrical appliances, and batteries serve as sources of Cu, Pb, and Cd attributing to the concentration of these elements in the indoor dust [4,5]. The high EF of Zn, Cu, Pb, and Ni indicates the presence of anthropogenic sources such as burning fossil fuels, paints, pigments, and metal alloys in household products and building materials [4]. The moderate EF of As and Cr indicates the enrichment of the metals from varied sources such as wood preservatives, chrome pigments, paints, ink, and anti-corrosive materials [4].

The As, Cr, Cu, and Fe spatial enrichment maps of the study region (Figure 6) demonstrates that the western and eastern parts of the Houston metropolitan region were more heavily enriched compared to others. The enrichment of Zn, Mn, and Ni were more pronounced in the western and northwestern regions while the Pb was seen distributed uniformly throughout the study area (Figure 7). Metal enrichment can be attributed to the various anthropogenic sources such as fossil fuels, industries, along with the individual household facilities and activities [4].

Health risk assessment revealed that ingestion can be a major pathway for Cd, Cr, and Ni exposure in children (Table 5). However, ingestion is the primary pathway of children exposure to indoor dust borne metals over inhalation and dermal contact [39]. Unfortunately, children were found to be more prone to long-term cancer risks from heavy metal exposure than the adults [39]. There were significant carcinogenic risks (TLCR > 10^−4^) from heavy metals in dust samples with Cd, Cr, and Ni being the main contaminants posing carcinogenic risks to children (Table 5).

In our samples, we observed multiple *Bacillus* spp. present. *Bacillus*, *Brevibacillus*, *Paenibacillus*, *Paenisporosarcina*, and *Sporosarcina* genera are all aerobic, spore-forming bacteria that can withstand industrial pasteurization and form biofilms within pipes and stainless-steel material. These single or multiple-species biofilms become a reservoir of spoilage microorganisms, and a sequence of contamination can be initiated [40,41]. Since we observed elevated *Bacillus* spp. in our dust samples, foodborne illness can become a major health risk because of the *Bacillus* spore-forming bacteria that present spoilage [42].

## 5. Conclusions

The spatial distribution of indoor dust borne contaminants and risk assessments from exposure to heavy metals in household dust was investigated for the Houston metropolitan region. Concentrations of Cd, Cu, Ni, Pb, and Zn in indoor dust were greater than background levels naturally occurring in Texas soils indicating anthropogenic influences. The enrichment of Cu and Zn was significantly higher compared to other elements in indoor dust samples. The health risk assessment revealed that ingestion was the major exposure route of metal, with children under six years old being the most vulnerable. SEM studies revealed varied particle shape, sizes, and elemental composition of indoor dust. Several Gram-positive and -negative bacteria were identified in representative dust samples. Gram-positive bacteria including *Bacillus* spp. can form persistent endospores and promote food poisoning and respiratory problems, while Gram-negative bacteria can cause respiratory disorders, pneumonia, womb infections, and gastric inflammation [43]. 

The limitation of the study includes that (1) the household attributes data collected were self-reported and hence resulted in poor correlation with the metal concentrations in the dust samples, (2) the effect of seasonal changes on indoor dust was not evaluated, and (3) the sampling residences were randomly selected by convenience and therefore may not represent the overall housing conditions in the region. Additionally, smoking frequency and home ventilation data were not collected as part of the survey. Indoor smoking can contribute significant metal contamination [44]. Results of this study highlight the importance of different metal contaminants and bacteria which can accumulate in indoor dust and pose risks to human health. Further research is needed to better understand the effect of seasonal changes on the chemical and bacterial contaminants.

## Figures and Tables

**Table 1:** Heavy metal concentrations in house dust samples collected from several locations within southeast Texas (in mg kg^−1^). Given are mean values (*n* = 3) of three replicates.

Habitat	Al	As	Cd	Cr	Cu	Fe	Ni	Pb	Zn
***Mean***	3738	3.6	1.9	23	53	2939	12	38	368
***Home Type***									
Apartment (*n* = 7)	5103 a	2.3	1.6	26	35 b	1304	9.4	32	221
Single Family (*n* = 24)	3179 b	4.1	2.1	22	61 a	3608	14	41	428
***Home Age***									
Under 10 years (*n* = 4)	3687	3.0	1.8 ab	29	56.5	8254	13	40 ab	464
10 to 30 years (*n* = 17)	3176	2.5	1.2 b	21	46.4	1930	11	25 b	297
Over 30 years (*n* = 10)	4662	5.6	3.2 a	24	62.6	1896	14	58 a	434
***Floor Type***									
Carpet (*n* = 14)	3805	4.3	1.6	24	38 b	1674	10	42 ab	241 b
Partial carpet (*n* = 12)	3507	2.8	1.7	21	67 a	4752	13	22 b	390 ab
No carpet (*n* = 5)	4102	3.5	3.3	25	65 a	2131	17	64 a	673 a
***Pets***									
No (*n* = 20)	3195 b	3.0	1.4	24	53	3507	12	34	333
Yes (*n* = 11)	4725 a	4.6	2.9	23	54	1907	12	45	432
***Heating***									
Electric (*n* = 16)	4103	2.4	1.2 b	22	48	1682	11	28	254
Gas (*n* = 15)	3348	4.8	2.7 a	25	59	4280	14	49	491
***^1^ Background***	30,000	5.9	0	30	15	15,000	10	30	15
***^2^ ESV***	N/A	18	0.36	23	28	N/A	38	11	46

Note: Values in the same column followed by letters a & b are significantly different at *p* < 0.05. ^1^ Texas-specific soil background concentrations [19]. ^2^ Region 4 Soil Screening Values for Hazardous Waste Sites [28]; ESV: Ecological Screening value.

**Table 2:** Elemental (in mg kg^−1^) and bacterial concentration (in cfu g^−1^) in house dust samples collected from several locations within southeast Texas. Given are mean values (*n* = 3) of three replicates.

Habitat	Na	Mg	K	Ca	Mn	TB	EB
***Mean***	17,387	2900	2872	8030	48.2	47,714	11,833
***Home Type***							
Multi-family (*n* = 7)	9868	2858	2040	4415	33 b	21,100	4733 b
Single-family (*n* = 24)	20,463	2917	3212	9508	54 a	58,360	14,673 a
***Home Age***							
Under 10 years (*n* = 4)	9300	2632	4125	4282	66	94,750 a	14,750
10 to 30 years (*n* = 17)	24,178	3063	2823	9709	45	35,227 b	10,909
Over 30 years (*n* = 10)	10,566	2773	2324	7217	45	39,250 ab	11,583
***Floor Type***							
Carpet (*n* = 14)	20,306	3249	2850	6104	41	60,618	13,636
Partial carpet (*n* = 12)	14,507	2859	3235	11,634	55	35,625	10,313
No carpet (*n* = 5)	16,128	2021	2062	4771	51	25,100	8000
***Pets***							
No (*n* = 20)	19,714	3038	2644	10,520	50	36,192	10,362
Yes (*n* = 11)	13,157	2649	3286	3502	44	66,438	14,225
***Heating***							
Electric (*n* = 16)	20,535	2875	2401	6096	41	53,810	9000
Gas (*n* = 15)	14,031	2927	3374	10,092	56	42,173	14,409
***^1^ Background***	N/A	N/A	N/A	N/A	300	N/A	N/A
***^2^ ESV***	N/A	N/A	N/A	N/A	220	N/A	N/A

Note: Values in the same column followed by letters a & b are significantly different at *p* < 0.05. ^1^ Texas-specific soil background concentrations [19]. ^2^ Region 4 Soil Screening Values for Hazardous Waste Sites [28], ESV: Ecological Screening value.

**Table 3:** Pearson correlation coefficients among elemental contaminants (*n* = 31) showing moderate (r = 0.40–0.69), strong (r = 0.70–0.89) and very strong (r = 0.90–1.0) correlations [18].

	Al	As	Cd	Cr	Cu	Fe	Ni	Pb	Zn	Na	Mg	K	Ca	Mn	TB
**As**	0.42 *														
**Cd**	0.36 *	0.54 **													
**Cr**	0.65 **	0.57 **	0.38 *												
**Cu**	−0.16	0.33	0.28	0.08											
**Fe**	−0.20	0.36 *	0.26	0.32	0.57 **										
**Ni**	0.09	0.41 *	0.15	0.27	0.61 **	0.62 **									
**Pb**	0.54 **	0.59 **	0.56 **	0.56 **	0.26	0.26	0.36 *								
**Zn**	−0.06	0.38 *	0.37 *	0.25	0.70 **	0.78 **	0.67 **	0.39 *							
**Na**	−0.47 **	−0.38 *	−0.27	−0.59 **	0.07	−0.23	−0.05	−0.50 **	−0.30						
**Mg**	0.06	0.23	−0.24	0.19	−0.23	0.18	−0.07	−0.04	−0.11	−0.31					
**K**	−0.11	0.29	0.15	0.21	0.42 *	0.48 **	0.38 *	0.00	0.37 *	0.31	0.04				
**Ca**	−0.52 **	−0.20	−0.33	−0.34	−0.06	0.2	−0.07	−0.31	−0.02	−0.15	0.53 **	−0.35			
**Mn**	−0.05	0.29	0.20	0.32	0.38 *	0.85 **	0.57 **	0.34	0.63 **	−0.38 *	0.29	0.31	0.29		
**TB**	0.19	0.45 *	−0.08	0.40	0.40	0.38	0.43 *	0.42	0.347	−0.18	0.10	0.46 *	−0.30	0.38	
**EB**	0.15	0.56 **	0.13	0.36	0.55 **	0.44 *	0.54 **	0.515 *	0.45 *	−0.06	0.03	0.56 **	−0.25	0.42	0.88 **

Note: Levels of significance: * *p* < 0.05, ** *p* < 0.01, The moderate to very strong correlations are underlined in the table.

**Table 4:** Averaged elemental weight percentages obtained from SEM-EDS, X-ray analysis for the selected dust samples. The SEM particle numbers correspond to the numbers displayed in the SEM micrographs (Figure 4).

SEM Particle	C	O	Na	Al	Si	S	Cl	K	Ca
Fiber (1)	49.9	34.1	3.4	0.6	2.3	1.0	2.4	1.1	1.8
Skin (2)	60.2	28.4	2.1	0.4	1.6	1.3	1.5	1.1	2.1
Sand (3)	69.9	10.3	1.5	1.6	10.2	1.3	1.5	0.9	2.0
Ca Salt (4)	35.5	40.2	0.7	0.6	1.6	9.1	0.9	0.5	10.8
Na Salt (5)	15.9	55.7	21.2	0.5	1.4	0.5	0.6	0.5	0.7

**Table 5:** Carcinogenic risk of priority heavy metals in dust from Harris County (*n* = 22), Brazoria County (*n* = 4), Fort Bend County (*n* = 4), and Montgomery County (*n* = 1) for children under 6 years of age. The underlined values indicate the higher cancer risk.

County	Risk	As	Cd	Cr	Ni
Harris	LCR ingestion	8.59 × 10^−5^	4.42 × 10^−4^	1.67 × 10^−4^	1.61 × 10^−4^
	LCR dermal	2.01 × 10^−6^	1.03 × 10^−5^	3.91 × 10^−6^	3.77 × 10^−6^
	LCR inhalation	5.41 × 10^−10^	2.78 × 10^−9^	1.05 × 10^−9^	1.02 × 10^−9^
	TLCR	8.79 × 10^−5^	4.52 × 10^−4^	1.71 × 10^−4^	1.65 × 10^−4^
Brazoria	LCR ingestion	3.58 × 10^−5^	2.03 × 10^−4^	1.33 × 10^−4^	1.30 × 10^−4^
	LCR dermal	8.37 × 10^−7^	4.74 × 10^−6^	3.12 × 10^−6^	3.04 × 10^−6^
	LCR inhalation	2.26 × 10^−10^	1.28 × 10^−9^	8.41 × 10^−10^	8.21 × 10^−10^
	TLCR	3.67 × 10^−5^	2.08 × 10^−4^	1.37 × 10^−4^	1.33 × 10^−4^
Fort Bend	LCR ingestion	2.56 × 10^−5^	3.13 × 10^−4^	1.12 × 10^−4^	9.30 × 10^−5^
	LCR dermal	5.97 × 10^−7^	7.31 × 10^−6^	2.61 × 10^−6^	2.17 × 10^−6^
	LCR inhalation	1.61 × 10^−10^	1.97 × 10^−9^	7.05 × 10^−10^	5.86 × 10^−10^
	TLCR	2.62 × 10^−5^	3.20 × 10^−4^	1.15 × 10^−4^	9.52 × 10^−5^
Montgomery	LCR ingestion	8.77 × 10^−5^	1.18 × 10^−4^	1.58 × 10^−4^	1.98 × 10^−4^
	LCR dermal	2.05 × 10^−6^	2.76 × 10^−6^	3.68 × 10^−6^	4.62 × 10^−6^
	LCR inhalation	5.53 × 10^−10^	7.45 × 10^−10^	9.93 × 10^−10^	1.25 × 10^−9^
	TLCR	8.97 × 10^−5^	1.21 × 10^−4^	1.61 × 10^−4^	2.02 × 10^−4^

**Table 6:** Biochemical test and colony color of isolated unknowns. Environmental isolates were identified through gram staining, biochemical reactions, and the BIOLOG Microstation.

Sample	Colony Color	Ribotyping/BIOLOG	Gram Stain	Catalase	Oxidase
S1	White	*Bacillus* sp. MG2–11	+	+	+
S4	Yellow	*Sporosarcina koreensis*	+	+	−
	Milk White	*Bacillus megaterium* strain EN2	+	+	+
S6	Gray	*Bacillus bingmayongensis* strain SCSB-19	+	+	+
	Grey whitish	*Bacillus muralis* strain QT332	+	+	+
S7	Orange	*Arthrobacter* sp. strain N5	+	+	−
S8	Oyster white	*Brevibacterium* sp. strain A9	+	+	+
	Creamy	*Bacillus oceanisediminis* strain NFS-CAP-3	+	+	+
S9	White	*Bacillus aryabhattai* strain G3	+	+	+
S10	Yellow-orange	*Bacillus firmus* strain S2	+	+	+
S11	Yellow	*Lysinibacillus sphaericus* strain D9	+	+	+
S12	Milk White	*Bacillus megaterium* strain EN2	+	+	+
S15	Creamy-Yellowish	*Bacillus koreensis* strain EFBL-YM2	+	+	+
	Orange	*Chryseobacterium* spp.	−	+	+
S24	Gray	*Aerococcus* spp. strain DE018	+	−	−
	Creamy	*Klebsiella aerogenes*	−	−	−
S25	Milk White	*Bacillus megaterium* strain TSM3	+	+	+
S28	White	*Bacillus megaterium* strain TSM3	+	+	+
S30	Yellow	*Pantoea* sp. strain JZ108	−	+	−

## Data Availability

Not applicable.

## Supplementary Materials

The following are available online at https://www.mdpi.com/article/10.3390/ijerph182312399/s1, Table S1: Residential dust sampling survey data.
